# Opioid use disorder and dementia risk: evidence from observational and genetic analyses in diverse ancestry cohorts

**DOI:** 10.1002/alz.71418

**Published:** 2026-05-22

**Authors:** Sara Javidnia, James M. Roe, Ville Karhunen, Dipender Gill, Steven Bell, Joseph D. Deak, Daniel Levey, Rachel L. Kember, Henry R. Kranzler, Héléne Toinét Cronjé, Stephen Burgess, Joel Gelernter, Klaus P. Ebmeier, Anya Topiwala

**Affiliations:** ^1^ Department of Psychiatry and Centre for Integrative Neuroimaging University of Oxford London UK; ^2^ Department of Radiology and Nuclear Medicine Computational Radiology and Artificial Intelligence Oslo University Hospital Oslo Norway; ^3^ Department of Psychology Center for Lifespan Changes in Brain and Cognition (LCBC) University of Oslo Oslo Norway; ^4^ MRC Biostatistics Unit, University of Cambridge Cambridge UK; ^5^ Department of Epidemiology and Biostatistics School of Public Health Imperial College London London UK; ^6^ Department of Oncology Precision Breast Cancer Institute University of Cambridge Cambridge UK; ^7^ Cancer Research UK Cambridge Centre Li Ka Shing Centre University of Cambridge Cambridge UK; ^8^ Department of Psychiatry Yale University School of Medicine New Haven Connecticut USA; ^9^ Veterans Affairs Connecticut Healthcare System West Haven Connecticut USA; ^10^ Department of Psychiatry University of Pennsylvania Perelman School of Medicine Philadelphia Pennsylvania USA; ^11^ Mental Illness Research Education and Clinical Center Crescenz Veterans Affairs Medical Center Philadelphia Pennsylvania USA; ^12^ Department of Public Health and Primary Care School of Clinical Medicine University of Cambridge Cambridge UK; ^13^ Big Data Institute Nuffield Department of Population Health University of Oxford Oxford UK

**Keywords:** dementia, Mendelian randomization, neurodegeneration, opioid use disorder, polygenic risk score

## Abstract

**INTRODUCTION:**

Opioid use disorder (OUD) may adversely affect brain health, but its role in dementia risk remains poorly understood.

**METHODS:**

We investigated associations between OUD and dementia using observational data from 222,518 participants (European and African ancestry) in the Million Veteran Program and Mendelian randomization (MR) using genome‐wide association study summary statistics from 6,066,918 individuals. Polygenic risk score (PRS) analyses were conducted in 229 opioid‐naïve Lifebrain consortium participants with longitudinal magnetic resonance imaging data.

**RESULTS:**

OUD was associated with increased risk of all‐cause dementia (hazard ratio = 1.56, 95% confidence interval [CI]: 1.39 to 1.76), Alzheimer's disease, and vascular dementia. MR supported a potential causal link between genetic liability to OUD and dementia (inverse variance weighted odds ratio = 1.77, 95% CI: 1.43 to 2.19). Genetic variation in the μ‐opioid receptor gene was also associated with dementia risk. No PRS associations were found with brain structure.

**DISCUSSION:**

These findings suggest a potential causal role for OUD in dementia, implicating μ‐opioid receptor pathways in neurodegeneration.

## Highlights

- Large‐scale observational study links OUD with increased dementia risk.
- MR supports a potential causal relationship between genetic liability to OUD and dementia.
- Genetic variation in the μ‐opioid receptor gene (*OPRM1*) is associated with dementia risk.
- No significant association was observed between OUD polygenic risk and brain structure in opioid‐naïve individuals.
- Findings highlight the need for further research into opioid‐related neurodegenerative mechanisms and long‐term brain health monitoring.

## 1 BACKGROUND

The opioid epidemic is a global public health crisis.^1^ While the short‐term consequences of opioid use and opioid use disorder (OUD) are well recognized,^2^, ^3^ their long‐term effects remain unclear. The latter are difficult to evaluate in randomized trials due to ethical constraints on prolonged opioid randomization, high attrition rates, limited follow‐up duration, and the frequent exclusion of individuals at highest risk. Emerging evidence suggests opioid use may accelerate cognitive decline,^4^, ^5^ highlighting the urgent need to clarify its potential long‐term neurodegenerative consequences as populations age and dementia prevalence rises.^6^, ^7^, ^8^

Observational studies suggest that individuals with a history of opioid use are at increased risk of cognitive impairment and dementia, with follow‐up durations ranging from a few years to approximately a decade across studies.^9^, ^10^, ^11^, ^12^, ^13^ Such associations are biologically plausible. Opioids act primarily through mu (μ‐) and delta (δ‐)opioid receptors (encoded by *OPRM1* and *OPRD1*), widely expressed in cognition‐related regions such as the hippocampus and cortex.^14^ Receptor activation modulates neurotransmitter release and key signaling pathways for neuronal communication,^15^, ^16^ with μ‐opioid receptor promoting microglial‐driven neuroinflammation and δ‐opioid receptor regulating inflammatory responses via microglial and astrocytes.^17^, ^18^ These processes contribute to synaptic dysfunction and neuronal injury, central mechanisms in dementia pathogenesis.^19^ However, evidence on neurodegenerative sequelae of opioid dependence remains limited, particularly in large, diverse, and older populations. In addition, causal inference is complicated by reverse causality and residual confounding related to socioeconomic factors^20^ and comorbidities such as chronic pain and psychiatric disorders,^21^ which are difficult to fully capture in observational data.^16^, ^22^

To address these limitations, we undertook a multi‐method investigation of the relationship between OUD and adverse brain health outcomes, including dementia, stroke, and brain structure. In this study, OUD is defined as a clinical diagnosis reflecting compulsive opioid use and loss of control and is conceptually distinct from prescription opioid exposure or intermittent opioid use for pain management. We first analyzed electronic health record data from the US Million Veteran Program (MVP) to assess observational associations between OUD and incident dementia in a large, ancestrally diverse population with extended follow‐up. We then conducted Mendelian randomization (MR) analyses using summary statistics from multiple genome‐wide association studies (GWASs), including UK Biobank (UKB) imaging‐derived phenotypes (IDPs), to strengthen causal inference and reduce confounding and reverse causation.^23^ Genetic liability to OUD reflects lifelong vulnerability to addiction‐related traits and behaviors, allowing separation of predispositional risk from effects that arise specifically from opioid exposure. To gain mechanistic insight, we performed cis‐MR analyses restricted to opioid receptor gene regions (*OPRM1* and *OPRD1*) and Bayesian colocalization to assess shared genetic architecture, approaches that reduce horizontal pleiotropy and strengthen causal interpretation.^24^ Finally, to disentangle genetic liability from opioid exposure, we evaluated whether polygenic risk for OUD was associated with longitudinal brain structural change among non‐opioid users in the Lifebrain cohort. Analyses of genetic liability to OUD and brain structure were designed to assess shared genetic architecture and predisposing neurobiological vulnerability, independent of the downstream effects of opioid exposure. This integrated approach provides complementary epidemiological and mechanistic evidence on the relationship between OUD and brain health.

## 2 METHODS

### 2.1 Study samples

**RESEARCH IN CONTEXT**

1. **Systematic review**: We reviewed existing literature on OUD and cognitive outcomes, including dementia. While prior studies suggested associations between opioid exposure and cognitive impairment, few addressed causality, ancestry differences, or long‐term neurodegenerative outcomes. To our knowledge, this is the first study to integrate observational data, MR, and polygenic risk analyses to assess the impact of OUD on dementia risk.
2. **Interpretation**: Our findings provide converging evidence from multiple methodologies that OUD is associated with increased risk of dementia, including Alzheimer's disease and vascular dementia. Genetic analyses support a potential causal relationship, implicating μ‐opioid receptor pathways. These results suggest that OUD may be a modifiable risk factor for dementia.
3. **Future directions**: Further studies are needed to explore the neurobiological mechanisms linking chronic opioid exposure to neurodegeneration, assess generalizability across global populations, and inform strategies for cognitive monitoring and dementia prevention in individuals with OUD.

For observational analyses, we included 222,518 unrelated MVP participants (193,744 of European [EUR] ancestry; 28,774 of African [AFR] ancestry; mean age 61.5 [standard deviation {SD} 10.1] years) enrolled from 2011 onwards (Supplemental Materials 1).^25^

The genetic analyses comprised 6,066,918 EUR ancestry participants across nine GWAS cohorts (Table S1). To examine the polygenic risk score of OUD liability (OUD‐PRS) and longitudinal brain changes independent of opioid exposure, we used data from the Lifespan Changes in Brain and Cognition (LCBC) study within the Lifebrain project. Opioid‐free status was an existing inclusion criterion of the Lifebrain consortium; we leveraged this feature to assess associations between genetic liability to OUD and brain structure in the absence of direct opioid exposure. The final sample included 229 participants with quality‐controlled genetic and longitudinal imaging data (138 women; mean age 65.6 [SD, 14.3] years). Full details of PRS computation, imaging protocols, modeling of brain structural change, and covariate adjustment are provided in Supplemental Materials 2.^26^

### 2.2 Opioid use traits

In MVP, lifetime OUD was defined using International Classification of Diseases, Ninth and Tenth Revision (ICD‐9/10) codes in electronic health record diagnoses consistent with established clinical criteria and was treated as a distinct clinical construct, separate from prescription opioid exposure or intermittent opioid use for pain management (Table S2). Diagnostic confidence was increased by requiring OUD code ≥2 for outpatients or ≥1 for inpatients. Individuals without an OUD diagnosis were classified as controls.^27^

### 2.3 Outcome measurements

In MVP, the primary outcome was all‐cause dementia, given uncertainty about opioid effects on specific subtypes. Cases were identified by ICD codes in the EHR (Table S3).^28^, ^29^ Prevalent cases at enrolment were excluded to reduce reverse causation. Secondary outcomes included dementia subtypes (Alzheimer's disease and vascular dementia), stroke subtypes, and neuroimaging endophenotypes of dementia and addiction.

In the polygenic risk score (PRS) analysis (Lifebrain consortium), we examined longitudinal changes in brain regions commonly affected by neurodegeneration,^30^, ^31^ including the cortex, hippocampus, amygdala, white matter, accumbens, caudate, pallidum, and thalamus.

### 2.4 Covariates

Potential confounders and effect modifiers were identified from prior research (Supplemental Materials 3).^10^, ^32^

### 2.5 Genetic associations and instrument selection

Genetic associations with OUD were sought from a large‐scale GWAS of seven cohorts (EUR: 15,251 cases; 538,935 controls).^27^ To avoid sample overlap with MVP, this study was chosen over another large OUD GWAS.^33^ Three independent (*r* ^2^ < 0.1) single‐nucleotide polymorphisms (SNPs) reached genome‐wide significance (*p* < 5 × 10^−8^): rs79704991 and rs1799971 in *OPRM1*, and rs11372849 in *FURIN* (Table S4). Instrument strength (*F*‐statistics)^24^ and heterogeneity (Cochran's Q)^34^ were assessed. No genome‐wide significant SNPs were identified in AFR (5435 cases; 79,442 controls). For cis‐MR, variant selection was biologically rather than statistically motivated and focused on genes encoding established opioid targets (*OPRM1*, *OPRD1*, *OPRK1*).^35^ This strategy reduces horizontal pleiotropy and increases confidence that effects act through the protein of interest.^24^ Full details are provided in Supplemental Materials 4 and Table S5. Genetic associations with all‐cause dementia were obtained from a GWAS of 451,317 EUR ancestry participants (25,473 cases) in MVP.^28^ For reverse MR, genome‐wide significant all‐cause dementia SNPs were identified as instruments (Supplemental Materials 4).^28^ Additional GWAS outcomes included alcohol use,^36^ cannabis use disorder,^37^ chronic pain,^38^ Alzheimer's disease,^39^ vascular dementia,^40^ stroke phenotypes,^41^ and UK Biobank imaging‐derived brain structure endophenotypes (Table S6).^42^

### 2.6 Statistical analyses

#### 2.6.1 Observational analysis

Cox proportional hazards models assessed associations between OUD and incident dementia, stratified by ancestry and adjusted for potential confounders. Follow‐up was from enrollment (when covariates were measured) to dementia diagnosis, death, or last data collection (December 2019), whichever occurred first. Proportional hazards assumptions were tested with time interactions; violations were addressed by stratifying covariates of no interest. Associations are reported as hazard ratios (HRs) with 95% confidence intervals (CIs).

#### 2.6.2 Genetic analyses

Bidirectional two‐sample MR was used to estimate associations between genetically predicted OUD liability and brain health outcomes. The inverse variance weighted (IVW) method was the primary analysis, with MR‐Egger^43^, ^44^ and multivariable MR^45^ applied for sensitivity. Mendelian Randomization Pleiotropy RESidual Sum and Outlier was not feasible owing to the limited number of variants.^46^ MRlap (R package version 0.0.3.3) was used to assess and correct for potential bias from sample overlap by applying cross‐trait linkage disequilibrium (LD) score regression to approximate overlap and generate bias‐corrected causal estimates,^47^ which were compared with standard IVW estimates. Reverse MR tested whether genetic liability to all‐cause dementia increased the risk of OUD liability. Analyses were conducted using the TwoSampleMR (version 0.6.3) and MendelianRandomization (version 0.9.0) R software packages. Variant harmonization ensured associations between SNPs and exposure/outcomes reflected the same allele. MR estimates were scaled to represent odds ratio (OR) change per doubling in OUD prevalence for interpretability.^48^ Standard MR analyses were conducted to provide etiological insights. Further analyses addressed specific pathways: (1) cis‐MR, restricting genetic instruments to those within opioid receptor genes (*OPRM1* and *OPRD1*) to reduce horizontal pleiotropy and strengthen causal inference; (2) Bayesian colocalization (version 5.2.3) to evaluate whether associations reflected shared causal variants, thereby reducing confounding by LD and providing stronger evidence for causality (Supplemental Materials 5)^49^, ^50^; and (3) PRS analyses in non‐opioid users from Lifebrain, testing whether OUD genetic liability was associated with accelerated brain atrophy using linear models.^26^

This study was conducted and reported in accordance with the Strengthening the Reporting of Observational Studies in Epidemiology‐MR guidelines for MR studies.

## 3 RESULTS

### 3.1 Observational analyses

Among 222,518 MVP participants, 9399 had a diagnosis of OUD (Tables S7 and S8). During follow‐up of up to 9 years (mean, 4.3 years); 7370 EUR and 1027 AFR participants developed all‐cause dementia. Individuals with OUD were younger at enrollment (mean age, 57.9 [SD, 10.8] years), had a higher proportion of males (91.3%), and were more likely to be daily smokers (40.6%) compared with those without OUD. They were also overrepresented in lower education and income categories (Table 1). Comorbid alcohol (66.5%) and cannabis (30.2%) use disorders were more common in participants with OUD, particularly among AFR individuals (Table 1).

**TABLE 1:** Demographic and clinical characteristics by opioid use disorder status in European and African ancestry individuals.

	European ancestry participants		African ancestry participants	
	Controls *N* = 186,839 (96.4%)	Opioid use disorder cases *N* = 6905 (3.6%)	Controls *N* = 26,280 (91.3%)	Opioid use disorder cases *N* = 2494 (8.7%)
Male, *N* (%)	176,790 (94.6%)	6304 (91.3%)	23,881 (90.9%)	2337 (93.7%)
Age, years (mean [SD])	66.9 (11.5)	57.91 (10.8)	61.8 (10.3)	59.03 (7.5)
Alcohol use disorder, *N* (%)	36,811 (19.7%)	4588 (66.5%)	9233 (35.1%)	2159 (86.6%)
Cannabis use disorder, *N* (%)	6069 (3.3%)	2083 (30.2%)	2905 (11.1%)	1165 (46.7%)
Smoking^a^, daily, *N* (%)	29,141 (15.6%)	2802 (40.6%)	5955 (22.7%)	987 (39.6%)
Education level^a^, *N* (%)				
Less than high school	7036 (3.8%)	287 (4.2%)	1273 (4.8%)	130 (5.2%)
Some college credit, but no degree	59,546 (31.9%)	2653 (38.4%)	9701 (36.9%)	1078 (43.2%)
Bachelor's degree (e.g., BA, BS)	32,198 (17.2%)	818 (11.9%)	3012 (11.5%)	163 (6.5%)
Professional/doctorate degree	5609 (3.0%)	120 (1.7%)	403 (1.5%)	13 (0.5%)
Income level^a^				
Less than $10,000	8305 (4.4%)	988 (14.3%)	3406 (13.0%)	582 (23.3%)
$10,000 to $19,999	28,131 (15.1%)	1678 (24.3%)	5538 (21.1%)	771 (30.9%)
$30,000 to $39,999	27,490 (14.7%)	890 (12.9%)	3549 (13.5%)	293 (11.8%)
$50,000 to $59,999	18,423 (9.9%)	508 (7.4%)	2123 (8.1%)	114 (4.6%)
$75,000 to $99,999	16,207 (8.7%)	349 (5.0%)	1603 (6.1%)	51 (2.0%)
$150,000 or more	4766 (2.6%)	64 (0.9%)	352 (1.3%)	12 (0.5%)
Dementia diagnosis, *N* (%)	7059 (3.8%)	311 (4.5%)	926 (3.5%)	101 (4.0%)

^a^ For brevity, only selected categories are presented.

**FIGURE 1:**
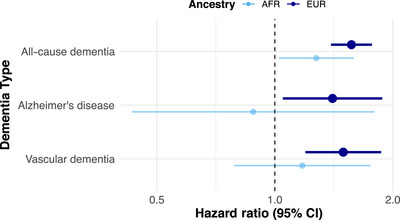
Opioid use disorder (OUD) and risk of dementia by ancestry. Observational associations between OUD and dementia subtypes in the Million Veteran Program (*n* = 222,518), stratified by European (EUR) and African (AFR) ancestry. Dots represent hazard ratios and lines their 95% confidence intervals. Estimates were generated using Cox proportional hazards regression. Models were adjusted for: age, sex, educational qualification, income, smoking status, alcohol use disorder, cannabis use disorder, post‐traumatic stress disorder symptoms, history of head injury, diabetes, and body mass index.

In Cox proportional hazards models, participants with a diagnosis of OUD had a significantly higher incidence of all‐cause dementia in both EUR (HR = 1.56, 95% CI: 1.39 to 1.76, *p* = 2.23 × 10^−13^) and AFR ancestry groups (HR = 1.27, 95% CI: 1.02 to 1.59, *p* = 0.03) (Figure 1), after adjustment for all identified confounds. Significant positive associations with OUD were also observed in the EUR group for Alzheimer's disease (HR = 1.40, 95% CI: 1.04 to 1.87, *p* = 0.02) and vascular dementia (HR = 1.49, 95% CI: 1.19 to 1.86, *p* = 4 × 10^−4^), unlike in the lower‐powered AFR analyses (Figure 1).

### 3.2 Genetic analyses

**FIGURE 2:**
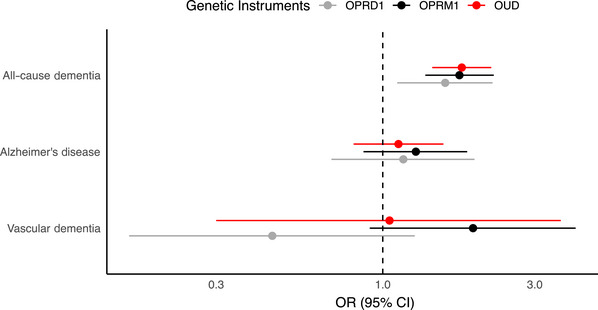
Genetic associations of opioid use disorder (OUD) and opioid receptor genes with dementia. Results are shown with 95% confidence intervals (CIs) and *p* values in corresponding colors: OUD in red, *OPRM1* in black, and *OPRD1* in gray for all‐cause dementia, Alzheimer's disease, and vascular dementia separately. Estimates were generated using the inverse variance weighted method and scaled to reflect genetic variants associated with a doubling of OUD prevalence. Source genome‐wide association studies used to derive genetic associations are described in Table S6.

Three independent genome‐wide significant variants that were associated with OUD were used as instruments in the primary MR analysis. All SNPs had strong instrument strength (*F‐*statistics 32.6 to 37.4; mean 34.9; Table S4), indicating low risk of weak instrument bias. Higher genetic propensity to OUD was significantly associated with all‐cause dementia (IVW OR = 1.77, 95% CI: 1.43 to 2.19, *p* = 1.69 × 10^−7^) (Figure 2 and Figure S1). Genetic estimates can be interpreted such that a doubling in OUD prevalence liability is associated with a 77% higher risk of all‐cause dementia. Robust methods gave broadly similar results (Table S9). Genetic associations remained significant in multivariable MR adjusting for genetically proxied chronic pain (Multi‐variable Mendelian randomization [MVMR] IVW OR = 1.82, 95% CI: 1.18 to 2.80, *p* < 0.01), alcohol use (MVMR IVW OR = 1.81, 95% CI: 1.41 to 2.33, *p* < 0.01), and cannabis use disorder (MVMR IVW OR = 1.87, 95% CI: 1.35 to 2.61, *p* < 0.01) (Table S10). Correction for sample overlap using MRlap did not materially alter the association between OUD liability and dementia (genetic correlation, *rg* = 0.033; SE = 0.0005; observed *β* = −0.0139; corrected *β* = −0.0210; *p* for difference = 0.99; Table S11). Reverse MR using four genome‐wide significant dementia variants as instruments showed no evidence that dementia liability influenced OUD risk (IVW *β* = −0.01, 95% CI: −0.46 to 0.44; *p* = 0.96; Figure S2). There was no evidence of heterogeneity across instruments (Cochran's Q IVW, *p* = 0.89; Table S12), or directional pleiotropy (MR‐Egger intercept, *p* = 0.83).

In cis‐MR analysis restricted to those genetic instruments within opioid receptor genes, we identified two independent variants in *OPRM1* (rs1799971 and rs79704991) and two in *OPRD1* (rs529520 and rs2236861). Instruments for both *OPRM1* (IVW OR = 1.75, 95% CI: 1.36 to 2.23, *p* = 9.5 × 10^−6^) and *OPRD1* (IVW OR = 1.57, 95% CI: 1.11 to 2.21, *p* = 0.01) were associated with increased all‐cause dementia risk (Figure 2 and Figure S3). Colocalization analysis (Figure S4) indicated that a shared genetic association signal between *OPRM1* instruments (PP.H0 = 1.25 × 10^−4^, PP.H1 = 0.06, PP.H2 = 1.88 × 10^−5^, PP.H3 = 0.01, PP.H4 = 0.93) and dementia was the most likely scenario. In contrast, a low likelihood of a shared causal variant between *OPRD1* instruments (PP.H0 = 0.35, PP.H1 = 0.40, PP.H2 = 0.01, PP.H3 = 0.01, PP.H4 = 0.22) and dementia was found, which may be attributable to low statistical power.

We performed secondary MR analyses of genetically proxied OUD propensity and different dementia subtypes, as well as relevant endophenotypes of dementia. Estimates were directionally concordant for both Alzheimer's disease (IVW OR = 1.12, 95% CI: 0.81 to 1.55, *p* = 0.52) and vascular dementia (IVW OR = 1.05, 95% CI: 0.30 to 3.62, *p* = 0.93), but with wide confidence intervals (Figure 2). See Table S9 for results using robust methods. Restricting genetic instruments to variants within *OPRM1* did not substantially alter associations (Figure S5). There were no significant associations with any stroke phenotype using the genome‐wide SNPs (stroke IVW OR = 1.04, 95% CI: 0.70 to 1.54, *p* = 0.84; ischemic stroke IVW OR = 1.07, 95% CI: 0.70 to 1.63, *p* = 0.74; cardioembolic stroke IVW OR = 0.68, 95% CI: 0.32 to 1.45, *p* = 0.31; large artery stroke IVW OR = 1.25, 95% CI: 0.21 to 7.31, *p* = 0.80; small vessel stroke IVW OR = 1.50, 95% CI: 0.77 to 2.90, *p* = 0.22) (Figures S6 and S7, Tables S13 and S14).

There were no significant genetic associations with any of the cross‐sectional GWAS‐derived phenotypes of neuroimaging, dementia, or addiction examined (Figure S8; see Figure S9 for more information), including the left hippocampus (IVW *β* = −0.22, 95% CI: −0.47 to 0.02, *p* = 0.07), left amygdala (IVW *β* = 0.05, 95% CI: −0.20 to 0.31, *p* = 0.68), and right ventral striatum (IVW *β* = 0.18, 95% CI: −0.07 to 0.43, *p* = 0.15) (Tables S15 and S16).

With respect to longitudinal brain changes using data from LCBC, in individuals without opioid exposure, no associations were observed between genetic propensity to OUD, estimated using either a stringent or more relaxed OUD PRS, and longitudinal brain atrophy. Regions tested included global cortical (PRS_relaxed_ *β* = 0.05, *t* = 0.8, *p* = 0.40) and subcortical regions, including the hippocampus (PRS_relaxed_ *β* = −0.05, *t *= −0.7, *p* = 0.40) and amygdala (PRS_relaxed_ *β* = −0.10, *t* = −1.6, *p* = 0.09) (Table S17).

## 4 DISCUSSION

In this large‐scale study, we triangulated research designs to investigate potential causal links between OUD liability and dementia, as well as the underlying biological mechanisms. Observational and genetic analyses supported a role for OUD liability in increasing dementia risk. Genetic evidence highlighted a potential etiological role for perturbation of the μ‐opioid receptor.

Associations observed between OUD and dementia in the present study are consistent with some prior reports,^9^, ^11^, ^12^, ^22^, ^51^ but not all.^52^, ^53^, ^54^ Differences across studies likely reflect important methodological heterogeneity. Some large studies examined prescription opioid use for non‐cancer pain rather than OUD and excluded individuals with addiction diagnoses. In these settings, elevated dementia risk was observed primarily at higher cumulative doses.^9^, ^12^ Other studies focusing on clinically ascertained OUD, with longer follow‐up and larger numbers of incident dementia cases, reported higher dementia risk among individuals with OUD.^11^ In contrast, studies reporting null associations have often had smaller sample sizes, shorter follow‐up, or a focus on cognitive test performance rather than incident dementia, with effects, when present, generally confined to higher opioid doses or specific cognitive domains rather than dementia outcomes.^52^, ^53^, ^54^ Studies in chronic pain populations have also reported increased dementia risk among opioid‐exposed individuals, although confounding by indication remains a consideration.^51^ Together, these differences in exposure definition (opioid use vs OUD), dose, follow‐up duration, outcome ascertainment, and statistical power likely contribute to the mixed findings in the literature. In MR analyses, genetic propensity to OUD was significantly associated with dementia. This was further supported by restricting instruments to opioid receptor genes and by colocalization, which suggested the associations were unlikely to reflect genetic confounding and may instead represent downstream consequences of OUD. Reverse MR showed no evidence for an effect of genetically predicted dementia on OUD, supporting directionality. Finally, we found no genetic associations with brain structure in individuals with minimal or no opioid exposure, suggesting that dementia risk may be driven by direct opioid exposure rather than shared genetic predisposition.

A key innovation of this study is the investigation of etiological pathways through *OPRM1* and *OPRD1*, encoding the μ‐ and  δ‐opioid receptors. While previous studies explored the role of these receptors in the development of opioid dependence,^14^, ^55^, ^56^ none directly examined their individual connection to dementia. We observed the strongest associations with genetically proxied μ‐opioid receptor perturbation. As the main mediator of opioids’ analgesic effects,^56^, ^57^ the μ‐opioid receptor also influences neurotransmitter release, synaptic plasticity, and neuroinflammation.^17^

The mechanisms linking opioid use or dependence to dementia remain unclear. Chronic opioid exposure may contribute via atherosclerosis, ^58^ infection,^59^ respiratory depression with hypoxia,^60^, ^61^ and hypotension,^62^ all of which can reduce cerebral blood flow and increase ischemic risk. Although we found no significant association between OUD liability and stroke, previous studies suggested cerebrovascular consequences of opioid use^63^, ^64^ that could contribute to cognitive decline and dementia.^65^ Further investigation is needed to clarify these relationships. Although we focused on Alzheimer's disease and vascular dementia due to greater diagnostic reliability and statistical power, associations between OUD and other neurodegenerative subtypes (e.g., Lewy body dementia, Parkinson's disease/dementia, frontotemporal dementia) remain important questions that we plan to investigate as case numbers increase with longer follow‐up and as sufficiently powered GWASs of these disorders become available. Despite associations with dementia, we found no clear genetic links with brain structure in individuals with no or minimal opioid exposure, clarifying smaller observational studies that report structural alterations in chronic opioid users.^31^, ^66^, ^67^ Our findings suggest that effects may be limited to those with direct opioid exposure, rather than reflecting genetic propensity to OUD. The higher prevalence of opioid use in MVP may explain observed dementia associations, whereas in UKB, where opioid use is less common, no such brain structure associations were detected.

A major strength of this study is the integration of large‐scale observational and genetic data. Consistency across such complementary methods with distinct biases are consistent with a possible causal relationship. Additional strengths include the large and diverse MVP sample, use of GWASs, and examination of multiple dementia outcomes (all‐cause, Alzheimer's disease, vascular dementia, and relevant endophenotypes). Our MR instruments were strong (high *F*‐statistics), and the risk of horizontal pleiotropy appeared low, supported by cis‐MR restricted to *OPRM1* and *OPRD1* and by MR‐Egger and Cochran's Q tests. The cis‐MR design, focusing on variants directly related to opioid biology, further reduced pleiotropy concerns. *FURIN* variants were excluded from cis‐MR given their involvement in pathways outside opioid signaling. Colocalization analyses indicated that dementia associations were more likely driven by shared causal variants downstream of OUD rather than independent effects. We further extended the analysis with PRS in non‐opioid users, demonstrating the absence of a link between genetic liability to OUD and brain atrophy without opioid exposure. Together, these results strengthen causal inference and clarify potential mechanistic pathways linking opioid exposure and dementia risk.

Several limitations should be noted. The statistical power was greater in EUR than AFR ancestry participants, and we could not assess from the EHR moderating factors, such as OUD duration, specific opioids used, including whether they were prescription or illicit opioids, or route of administration. The MVP sample^25^ is predominantly male with military exposure and higher OUD prevalence, limiting generalizability. OUD and dementia diagnoses were derived from ICD codes, which may be misclassified or under‐recorded, biasing results toward the null and making observed associations conservative. Dementia may also be underdiagnosed in opioid users, as reversible cognitive effects could obscure recognition. Although we adjusted for comorbidities, socioeconomic factors, and other substance use disorders, residual confounding is possible; for instance, body mass index is an imperfect proxy for adiposity, especially in non‐European groups. Pain‐related conditions may contribute to OUD risk; however, chronic pain is poorly captured in EHR data, and the consistency of findings across observational analyses and multivariable MR accounting for genetic liability to chronic pain suggests that residual confounding by pain alone is unlikely to explain the observed associations. MR estimates are less affected by such confounding and capture lifelong genetically proxied OUD liability, distinct from later‐life opioid use.^24^, ^68^ Partial GWAS overlap could bias results toward observational associations, but MRlap correction indicated no material effect. Finally, *OPRD1* variants did not reach significance, unlike stronger *OPRM1* variants,^27^, ^33^, ^69^ which may explain why μ‐opioid receptor associations were clearer than δ‐opioid receptor associations. These differences likely reflect both their relative roles in OUD and the reduced statistical power of the *OPRD1* cis‐MR instrument.

Both observational and genetic evidence consistently supported an association between OUD liability and dementia risk. The μ‐opioid receptor (encoded by *OPRM1*) was implicated as a key biological component. These findings highlight the need for caution in prescribing opioids for long‐term use and call for enhanced pharmacovigilance. Future research should explore the biological mechanisms linking *OPRM1* to cognitive decline.

## CONFLICT OF INTEREST STATEMENT

D.G. is the Chief Executive Officer of Sequoia Genetics, a private limited company that collaborates with investors, pharmaceutical companies, biotech firms, and academic institutions to leverage genetic data for drug discovery and development. D.G. also holds interests in several biotechnology companies and has received consulting fees from Tourmaline Bio and QubeCo Bio. He holds stock of Apollo Therapeutics and RheumaLogics. H.T.C. is employed by Sequoia Genetics. H.K. is a member of advisory boards for Altimmune, Clearmind Medicine, Dicerna Pharmaceuticals, Enthion Pharmaceuticals, Lilly Pharmaceuticals, and Sophrosyne Pharmaceuticals; a consultant to Sobrera Pharmaceuticals and Altimmune; the recipient of research funding and medication supplies for an investigator‐initiated study from Alkermes; a member of the American Society of Clinical Psychopharmacology's Alcohol Clinical Trials Initiative, which was supported in the last 3 years by Alkermes, Dicerna, Ethypharm, Imbrium, Indivior, Kinnov, Lilly, Otsuka, and Pear; and an inventor on US provisional patent 63/710,270 “Multi‐ancestry Genome‐wide Association Meta‐analysis of Buprenorphine Treatment Response,” filed October 29, 2024. SBu consults for Sequoia Genetics on projects that do not overlap with this work. J.G. is paid for editorial work for the journal *Complex Psychiatry*. S.J., J.R., V.K., S.Be., J.D., D.L., R.K., K.P.E., and A.T. have nothing to disclose. Author disclosures are available in the Supporting Information.

## CONSENT STATEMENT

This study was conducted using data from the US Department of Veterans Affairs Million Veteran Program (MVP). All MVP participants provided written informed consent, and the program was approved by the VA Central Institutional Review Board. The current analysis was approved by the appropriate oversight bodies and complies with all relevant ethical regulations for human subject research. MR analyses were performed using publicly available, de‐identified GWAS summary statistics from previously published studies. All original studies had obtained ethics approval and participant consent, and no new data collection was undertaken for this component of the research.

## Supporting information



Supporting Information

Supporting Information

Supporting Information

Supporting Information
