# Supplementary material for: Opioid use disorder and dementia risk: evidence from observational and genetic analyses in diverse ancestry cohorts
Source: Alzheimers Dement. 2026 May 22;22(5):e71418. doi: 10.1002/alz.71418 (PMC13240559; doi:10.1002/alz.71418)
Supplement: Supplementary file 2 — Supporting Information [file ALZ-22-e71418-s003.docx]

# **Supplementary Materials**

## **Supplemental materials 1: MVP population for observational study**

## Recruitment was primarily conducted through invitational mailings and in-person enrolment at Department of Veterans Affairs (VA) facilities across the United States. Veterans who were active users of the Veterans Health Administration (VHA) and able to provide informed consent were eligible to participate. Data collection included questionnaires, blood samples for genomic testing, and access to participants’ VA electronic health records.^25^ For the present analyses, unrelated participants (kinship coefficient <0.088) were included. Broad ancestries were assigned using genetic data with reference to the 1000 Genomes Project panels.

## **Supplemental materials 2: Polygenic Risk Score Study population**

We computed PRS-OUD using both a stringent genome-wide significance threshold (p < 5×10⁻⁸) and a more relaxed threshold (p < 0.1). To address linkage disequilibrium, SNPs were pruned for near independence in PLINK (parameters: --clump-p1 0.9999, --clump-p2 0.9999, --clump-r2 0.1, --clump-kb 500), using European LD structure from the 1000 Genomes Project Phase 3. Participants were drawn from the Center for Lifespan Changes in Brain and Cognition (LCBC) at the University of Oslo, with MRI data collected on three scanners. Each participant contributed 2–7 imaging timepoints (median, 3; follow-up, 73 days to 11.1 years). All provided written informed consent, and the study was approved by relevant ethics committees.

Brain structural change was operationalized as individual-specific random slopes from generalized additive mixed models with a nonlinear age term, spanning the adult lifespan. Models adjusted for sex, scanner type, intracranial volume, and mean age across timepoints. For each brain metric, linear models tested associations between PRS-OUD and random slopes, with additional covariates for number of timepoints and the first 10 ancestry principal components to control for population structure. All participants had no prior opioid exposure. This design enabled testing whether genetic propensity to OUD was associated with longitudinal brain structural changes in healthy adults. Random slopes, which index individual deviation in age-related brain change, have been validated in similar datasets through correlation with Alzheimer disease polygenic scores,^26^ supporting their interpretability as markers of genetic influence.

## **Supplemental materials 3: Covariate measurement**

In the MVP, sociodemographic factors (age, sex, education, income), lifestyle behaviors (smoking), and physical and psychiatric health (body mass index, history of head injury, post-traumatic stress disorder, and diabetes) were assessed using self-reported enrolment and lifestyle questionnaires, supplemented by electronic health records (EHR). Education was classified into seven categories: less than high school, high school diploma, some college credit, associate's degree, bachelor’s degree, master’s degree, and professional or doctoral degree. Household income was recorded in nine ranges: less than $10,000; $10,000–19,999; $20,000–29,999; $30,000–39,999; $40,000–49,999; $50,000–59,999; $60,000–149,000; and $150,000 or more. Smoking status was categorized as daily, occasional, or not at all.

BMI was calculated from self-reported height and weight at enrolment. A history of head injury, PTSD, and diabetes mellitus was reported on enrolment surveys and coded as binary variables. Comorbid alcohol and cannabis use disorders were identified using ICD codes in the EHR. Mean systolic and diastolic blood pressure values were calculated from multiple EHR measurements.^25^

**Supplemental materials 4: Genetic associations and instrument selection**

Genetic associations with OUD were obtained from a large GWAS meta-analysis of seven cohorts (European ancestry: 15 251 cases; 538 935 controls; African ancestry: 5 435 cases; 79 442 controls).^27^ This study was chosen over an alternative OUD GWAS,^33^ which relied predominantly on the Million Veteran Program (MVP) and would have introduced substantial sample overlap with downstream analyses. In European ancestry, three independent (r² < 0.1) SNPs reached genome-wide significance (p < 5 × 10⁻⁸) and were selected as instruments for the primary MR analysis: rs79704991 and rs1799971 in *OPRM1*, and rs11372849 in *FURIN*. When rs11372849 was unavailable in a given dataset, the high-LD proxy rs17514846 (*FURIN*, r² > 0.8) was used. Instrument strength was evaluated using F-statistics,^24^ and heterogeneity across instruments was assessed with Cochran’s Q.

No genome-wide significant SNPs were identified in African ancestry.
For cis-MR, variant selection was biologically rather than statistically driven, with emphasis on genes encoding established opioid targets.^34^ This approach reduces horizontal pleiotropy and increases confidence that observed effects act through the protein of interest. Two genome-wide significant SNPs (rs1799971 and rs79704991) were located within *OPRM1* (μ-opioid receptor).^35^ Two weaker instruments were identified within *OPRD1* (δ-opioid receptor; rs529520, P = .001; rs2236861, P = 8 × 10⁻⁶) (Supplemental.T5).

Although *OPRK1*, which encodes the kappa (κ-)-opioid receptor, was investigated due to its relevance as an opioid target, no strongly associated variants were identified in this gene region.
Genetic associations with all-cause dementia were obtained from a GWAS of 451 317 participants of European ancestry (25 473 cases) in MVP.^28^ For reverse MR, four genome-wide significant dementia SNPs were identified as potential instruments: rs3851179, rs429358, rs111371860, and rs744373. Of these, rs3851179 and rs744373 were available in the OUD GWAS, whereas rs429358 and rs111371860 were not, and no suitable proxies could be identified.

**Supplemental materials 5: Bayesian colocalization**

Colocalization quantifies the posterior probability (PP) of hypotheses (H) given the data, including a shared (PP.H4) vs distinct (PP.H3) causal variant between the exposure and outcome at the exposure gene locus. A threshold of PP.H4 + PP.H3 > 0.5 and PP.H4 / (PP.H4 + PP.H3) > 0.5 was considered evidence of colocalizing signals. Conversely, PP.H3 + PP.H4 > 0.5 and PP.H4 / (PP.H4 + PP.H3) < 0.5 supports that the genetic variants influencing an exposure and an outcome are distinct, and MR estimates may be influenced by genetic confounding through variants in linkage disequilibrium. Other hypotheses tested included PP.H0, which represents the probability of no association with either trait; PP.H1, the probability of an association with the exposure only; and PP.H2, the probability of an association with the outcome only.^50^
